# Blood volume analysis as a guide for dry weight determination in chronic hemodialysis patients: a crossover study

**DOI:** 10.1186/s12882-019-1211-7

**Published:** 2019-02-11

**Authors:** Line Malha, Hasan Fattah, Frank Modersitzki, David S. Goldfarb

**Affiliations:** 1 Nephrology and Hypertension Division, Weill-Cornell Medicine, 424 East 70th street, New York, NY 10021 USA; 2 Nephrology Division, University of Kentucky, UK Transplant Center, 740 S. Limestone, 3rd fl, suite K348, Lexington, KY 40536 USA; 3 Nephrology Section, New York Harbor VA Healthcare System, Nephrology Section/111G, 423 East. 23 St., New York, NY 10010 USA; 4 Nephrology Division, NYU School of Medicine and NYU Langone Medical Center, New York University School of Medicine, 423 E. 23 St., New York, NY USA

**Keywords:** Volume control, Iodine radioisotopes/diagnostic use, Ultrafiltration, Volume status, Absolute blood volume; dry weight; hemodialysis

## Abstract

**Background:**

Volume overload and depletion both lead to high morbidity and mortality. Achieving euvolemia is a challenge in patients with end stage kidney disease on hemodialysis (HD). Blood volume analysis (BVA) uses radiolabeled albumin to determine intravascular blood volume (BV). The measured BV is compared to an ideal BV (validated in healthy controls). We hypothesized that BVA could be used in HD to evaluate the adequacy of the current clinically prescribed “estimated dry weight” (EDW) and to titrate EDW in order to improve overall volume status. We were also interested in the reproducibility of BVA results in end stage kidney disease.

**Methods:**

Twelve adults on chronic HD were recruited; 10 completed the study. BVA (Daxor, New York, NY, USA) was used to measure BV at baseline. EDW was kept the same if the patient was deemed to be euvolemic by BVA otherwise, the prescribed EDW was changed with the aim that measured BV would match ideal BV. A second BVA measurement was done 1–3 months later in order to measure BV again.

**Results:**

Based on BVA, 6/10 patients were euvolemic at baseline and 5/10 were euvolemic at the second measurement. When comparing patients who had their prescribed EDW changed after the initial BVA to those who did not, both groups had similar differences between measured and ideal BV (*P* = 0.75). BV values were unchanged at the second measurement (*P* = 0.34) and there was no linear correlation between BV change and weight change (r^2^ = 0.08).

**Conclusions:**

This pilot study is the first longitudinal measurement of BVA in HD patients. It revealed that changing weight did not proportionally change intravascular BV. BV remained stable for 1–3 months. BVA may not be helpful in clinically stable HD patients but studies on patients with hemodynamic instability and uncertain volume status are needed.

**Trial registration:**

ClinicalTrials.gov (NCT02717533), first registered February 4, 2015.

## Background

A major purpose of hemodialysis is to remove excess salt and water and restore extracellular fluid volume (ECV) to normal. Solute clearance has been widely used as an objective method to assess dialysis adequacy but there is no equivalent objective assessment for adequate and appropriate volume status. More recently, experts have promoted a “volume first approach”. A clear, objective method to assess ECV would be paramount to the success of this approach [1, 2].

Chronic volume overload is common in dialysis patients and is often unrecognized by physical exam [3, 4] leading clinicians to overestimate dry weight and underestimate the ultrafiltration requirement. Prior studies have demonstrated that in patients with end stage kidney disease (ESKD), volume expansion correlates with elevated blood pressure [5, 6] and adjusting the target weight leads to improved blood pressure control [7–10]. ECV overload, as determined by bioimpedance [11–14] and lung ultrasound [15], has also been associated with increased mortality and cardiovascular events in chronic kidney disease.

The benefit of additional ultrafiltration and decrease in estimated dry weight (EDW) [7], needs to be weighed against the risks of intradialytic hypotension that can also lead to adverse cardiovascular outcome and mortality [16–18].

There is currently no consensus about which objective measurement of ECV may provide clinically useful and accurate measurement of blood volume (BV) [19] to assist clinicians in finding a safe middle ground between intravascular volume depletion and deleterious ECV expansion. Blood volume analysis (BVA) using radiolabeled albumin is often considered the gold standard to determine intravascular blood volume (BV) and allows comparison to an “ideal” BV [20, 21] (as detailed in the methods section).

BVA has been used with some benefit to evaluate volume status in critically ill [22–25] as well as non-critically ill [26] hospitalized patients. We previously demonstrated that BVA could be used successfully in hemodialysis patients to determine BV, and changes in BV, before and after dialysis, with good correlation with relative BV changes as measured by the online Crit-Line Monitor (CLM III; Hema Metrics, Kaysville, UT, USA) [27].

More recently, Leung et al. have failed to demonstrate a significant difference in hemodialysis outcomes (including intradialytic hypotension) in a randomized clinical trial comparing 32 patient undergoing hemodialysis with and without relative BV monitoring [28]. The lack of benefit from relative BV monitor thus raises the question of the potential benefit of absolute BV measurement, a more accurate indicator of intravascular volume and thus, a more relevant monitoring technique for BV in dialysis patients [29].

In the current study we hypothesized that BVA could be further used in the dialysis unit to evaluate the adequacy of the current clinically prescribed EDW in dialysis patients and to titrate EDW in order to improve overall volume status and decrease the incidence of blood volume misinterpretation. In addition, we were interested in the reproducibility of BVA results in HD patients.

## Methods

### Patients

**Fig. 1:**
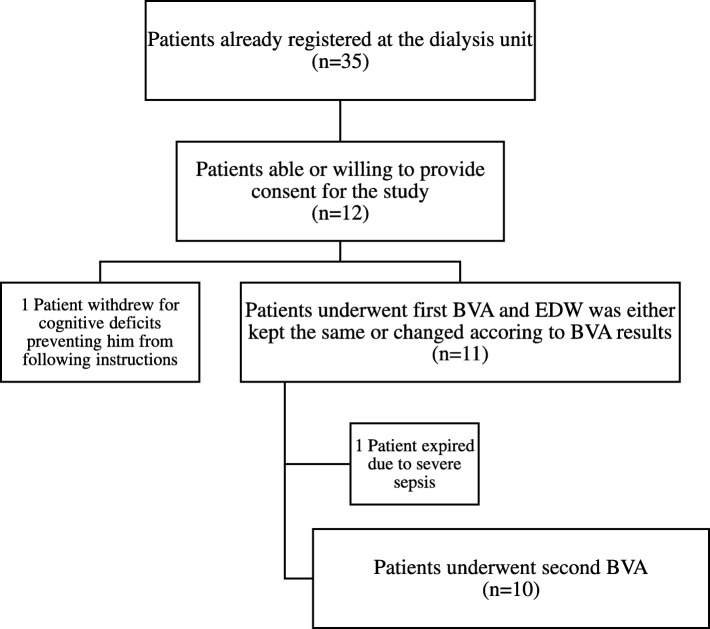
Participant flow chart. *BVA* blood volume analysis, *EDW* estimated dry weight

Eligible patients had ESKD receiving chronic, thrice weekly hemodialysis at the New York Harbor Department of Veterans Affairs Healthcare System (VA-NYHHS). Patients could be enrolled if they were at least 21 years old, able to give informed consent, and willing to have two blood volume measurements performed. We enrolled 12 eligible patients (Fig. 1) between 03/19/14 and 06/03/14. Participants underwent post-hemodialysis BVA as a baseline measurement. Based on their BV status, their prescribed EDW was adjusted (as detailed below). After 1 to 3 months, participants underwent a repeat BV measurement. We compared the second value to the first in order to judge whether an improvement in BV status occurred if the dry weight prescription was changed, or to judge stability if it was not.

Blockade of iodine uptake by the thyroid gland was achieved with a saturated solution of potassium iodide. Participants took 130 mg/day (1 drop of 1 g/ml potassium iodide solution in 8 oz of water) the day prior to the procedure and for 7 days after the BVA.

The study was approved by the local Institutional Review Board and registered at ClinicalTrials.gov (NCT02717533).

### Hemodialysis protocol

Hemodialysis was performed in accordance with standard, routine clinical care using the Phoenix® (Gambro, Stockholm, Sweden) delivery system. The dialyzers used were: Polyflux 210 (Gambro) or Rexeed 25SX (Asahi) as per the patient’s prior prescription. Blood flow rates were between 400 and 500 ml/min and a standard bicarbonate dialysate was used. The treatment time was individualized depending on urea reduction rates and ultrafiltration needs. Vital signs (including blood pressure and heart rate) were recorded at the beginning, during and at the end of the dialysis treatment as per standard practice. Pre- and post- dialysis weights were documented.

Information about intradialytic complications was collected including: intradialytic hypertension (increase in blood pressure ≥ 20 mmHg), intradialytic hypotension (systolic blood pressure < 90 mmHg or decrease in blood pressure ≥ 20 mmHg), tachycardia (heart rate 100/min, or change > 30/min), cramping and, symptoms of volume depletion (dizziness, weakness, nausea, pre-syncope or syncope) requiring medical intervention (interrupting treatment, adjusting ultrafiltration or administration of normal saline).

### Blood volume analysis

BVA was performed using the BVA-100 device (Daxor Corporation, New York, NY) applying the indicator dilution technique [30]. All BV measurements were made at the VA-NYHHS nuclear medicine area within an hour of ending the participants’ hemodialysis session. A blood sample was collected at baseline for evaluation of peripheral hemactocrit (Hct) and background radioactivity. Then, 25 microCuries of Iodine-131 labeled albumin were injected intravenously. After 12 min (in order to allow for proper mixing of radiolabeled albumin), 5 ml blood samples were drawn at 6 min intervals for a total of 6 samples. Hct was measured using HemataSTAT II® Microhematocrit System (STI, Sanford, FL) for all samples then; the radioactivity level was measured using the BVA-100 device. Linear regression for radioactivity level of serial samples reflects the transudation of fluid from the intravascular space to the interstitium and allows BV and plasma volume (PV) determination by extrapolation to volume of distribution at time zero.

BVA-100 device software calculates blood volume and plasma volume according to the equations below:

$$ PV=\frac{1000\ \mathrm{x}\ \left(\mathrm{standard}\ \mathrm{count}\hbox{-} \mathrm{baseline}\ \mathrm{count}\right)}{\left(\mathrm{sample}\ \mathrm{count}\hbox{-} \mathrm{baseline}\ \mathrm{count}\right)}\kern0.5em BV=\frac{\mathrm{PV}}{\left(1\hbox{-} \mathrm{Hct}\ \mathrm{x}\ 0.99\ \mathrm{x}\ 0.91\kern0.5em \frac{peripheral\kern0.5em Hct}{whole\kern0.5em body\kern0.5em Hct}\right).} $$

### Estimated dry weight adjustment

Ideal BV is estimated from reference curves derived from the standard Metropolitan Life Insurance Company Desirable Weight tables in order to take into account variability in body habitus [21, 30, 31]. For every patient, BV was measured at baseline and compared to the corresponding ideal BV value. The difference between ideal and measured BV allowed an estimation of the degree of hypervolemia or hypovolemia at baseline, and guided the prescription of EDW. The deviation from ideal BV was considered statistically significant when it is larger than ±8% as supported by the manufacturer’s manual and published literature [21]. The target EDW was defined as the post-dialysis (post-HD) weight at which patient’s post-dialysis BV would equal ideal BV. The patient’s EDW prescription was changed in order to achieve this target EDW. This definition assumes that ideal BV defines euvolemia for each participant and that 1 L of BV change would be equivalent to 1 kg of body weight. Subsequent changes to the prescribed EDW were made according to clinical indications, if needed.

### Statistical analysis

Statistical analysis was performed using Mann-Whitney U test, Wilcoxon signed rank test and Chi-square test when comparing independent samples. Fisher’s exact test was used when comparing 2 groups with less than 5 patients. For paired samples (comparing first to second measurements), Signed rank test was used. Statistical testing was computed using the Statistical Package for the Social Sciences (SPSS 20 (SPSS Inc., Chicago). A 2-tailed *p*-value < 0.05 was considered to reach statistical significance. Only patients who underwent both BVA measurements were included in the statistical analysis.

## Results

All patients, except for patient number 9, were men with baseline demographics described in Table 1. At baseline, only 3/10 patients were within 0.5 kg of prescribed EDW. Based on BVA and comparison to “ideal” BV, 6 of 10 patients were euvolemic at baseline (Table 2). Only 1 was judged to be volume overloaded and 3 were considered volume depleted at baseline. Age, blood pressure and heart rate were similar (*p* value > 0.05) for patients that required a change in EDW compared with those who did not.

**Table 1:** Patient characteristics

	Mean ± Standard Deviation
Age	40 ± 13
Years on dialysis	4.3 ± 3.2
Blood pressure (mmHg)	144/ 78 ± 28/ 10
Heart rate (beats/min)	82 ± 12
Deviation in BV (%)	−2.7 ± 9.8
Time interval between the 2 measurements (days)	70 ± 29
	N (%)
Gender (Male/Female)	9/1 (90/10)
Race	
Black	7 (70.0)
White	1 (10.0)
Unknown / Not Reported	2 (20.0)
Ethnicity	
Hispanic or Latino	2 (20.0)
NOT Hispanic or Latino	8 (80.0)
Was the EDW changed after initial BVA?	
No	4 (40.0)
Yes	6 (60.0)

**Table 2:** Individual patient clinical data

Patient	Age	EDW order changed	Interval between Measurements (days)	Overall BV at Baseline	Overall BV at Follow Up	% Deviation in BV from Ideal at Baseline	% Deviation in BV from Ideal at Follow Up	Post HD Weight at Baseline	Post HD Weight at Follow Up	Post HD BP Baseline	Post HD BP Follow Up	Intradialytic complications^a^at Baseline	Intradialytic complications^a^Follow Up
1	56	No	38	Euvolemic	Euvolemic	−1.1	2.7	86.0	86.4	155/86	160/72	None	None
2	69	No	41	Euvolemic	Volume overloaded	6.9	11.0	108.3	107.9	138/65	155/72	None	None
3	68	No	45	Euvolemic	Euvolemic	1.4	1.1	81.8	81.2	169/73	124/69	None	None
4	60	No	88	Euvolemic	Euvolemic	−5.2	6.5	71.1	75.3	153/76	150/77	None	Hypertension
5	84	Yes	32	Volume depleted	Volume depleted	−16.7	−19.3	60.4	61.9	187/87	143/86	None	Hypertension
6	58	Yes	98	Volume overloaded	Volume overloaded	11.1	22.6	57.7	57.1	162/85	187/101	None	Cramps and Tachycardia
7	60	Yes	84	Euvolemic	Euvolemic	8.0	7.8	85.9	86.5	147/101	162/90	Cramps	Cramps and Hypertension
8	52	Yes	119	Euvolemic	Euvolemic	−6.2	3.2	86.6	86.2	116/60	106/40	None	Cramps and Hypotension
9	44	Yes	75	Volume depleted	Volume overloaded	−8.4	8.6	107.9	109.5	118/63	134/88	None	None
10	83	Yes	80	Volume depleted	Volume depleted	−17.2	−38.3	92.0	92.1	134/61	156/76	None	Hypertension

*EDW* estimated dry weight prescribed, *BV* blood volume, *HD* hemodialysis, *BP* blood pressure,

^a^Intradialytic complications include: intradialytic hypertension (increase in blood pressure ≥ 20 mmHg), intradialytic hypotension (systolic blood pressure < 90 mmHg or decrease in blood pressure ≥ 20 mmHg), tachycardia (heart rate 100/min, or change > 30/min), cramping and, symptoms of volume depletion (dizziness, weakness, nausea, pre-syncope or syncope) requiring medical intervention

As per protocol, we then changed the EDW prescribed for the next hemodialysis for these 4/10 patients in order to bring post-HD weight near target EDW (Table 3). We also changed the EDW order for 2 patients who had BV measures suggesting that, although deemed “euvolemic”, they were further from ideal BV than might be considered desirable: patient 7 was above ideal BV by 8.0%, and patient 11 was below ideal BV by − 6.2%. These changes were consistent with the protocol and intended to optimize BV. Hemodialysis was then performed with the new target EDW, and BVA was repeated after a variable interval of 1–3 months (Table 1).

**Table 3:** Individual patient data detailing blood volume status, prescribed estimated dry weight and measured post-dialysis weight

Patient	EDW order changed	Overall BV at Baseline	Overall BV at Follow Up	%Deviation in BV from Ideal at Baseline	% Deviation in BV from Ideal at Follow Up	Post HD Weight at Baseline	Post HD Weight at Follow Up	Prescribed EDW Baseline	EDW prescribed after Baseline results	Prescribed EDW at Follow Up
1	No	Euvolemic	Euvolemic	−1.1	2.7	86.0	86.4	85.5	85.0	85.0
2	No	Euvolemic	Volume overloaded	6.9	11.0	108.3	107.9	108.0	107.5	107.5
3	No	Euvolemic	Euvolemic	1.4	1.1	81.8	81.2	81.0	81.5	81.5
4	No	Euvolemic	Euvolemic	−5.2	6.5	71.1	75.3	69.5	71.5	72.5
5	Yes	Volume depleted	Volume depleted	−16.7	−19.3	60.4	61.9	57.0	62.0	62.0
6	Yes	Volume overloaded	Volume overloaded	11.1	22.6	57.7	57.1	56.5	55.0	55.0
7	Yes	Euvolemic	Euvolemic	8.0	7.8	85.9	86.5	84.5	83.5	86.0
8	Yes	Euvolemic	Euvolemic	−6.2	3.2	86.6	86.2	86.0	87.0	85.0
9	Yes	Volume depleted	Volume overloaded	−8.4	8.6	107.9	109.5	108.0	109.5	108.0
10	Yes	Volume depleted	Volume depleted	−17.2	−38.3	92.0	92.1	90.5	94.5	92.0

*EDW* estimated dry weight prescribed, *BV* blood volume, *HD* hemodialysis, *BP* blood pressure

The difference between measured BV and ideal BV did not significantly differ from the first to the second BVA measurement (*P* value 0.75). Patients remained in the same volume status category whether their EDW was changed according to BVA or not (P value 0.04). BV remained unchanged at the second measurement (Correlation coefficient 0.81, P value 0.004). Among patients whose EDW was kept the same after the BVA measurement, 4/5 were judged to be euvolemic at follow up. The other patient, #2, had an increase in BV to 11% above ideal BV, resulting in a diagnosis of mild volume overload.

**Fig. 2:**
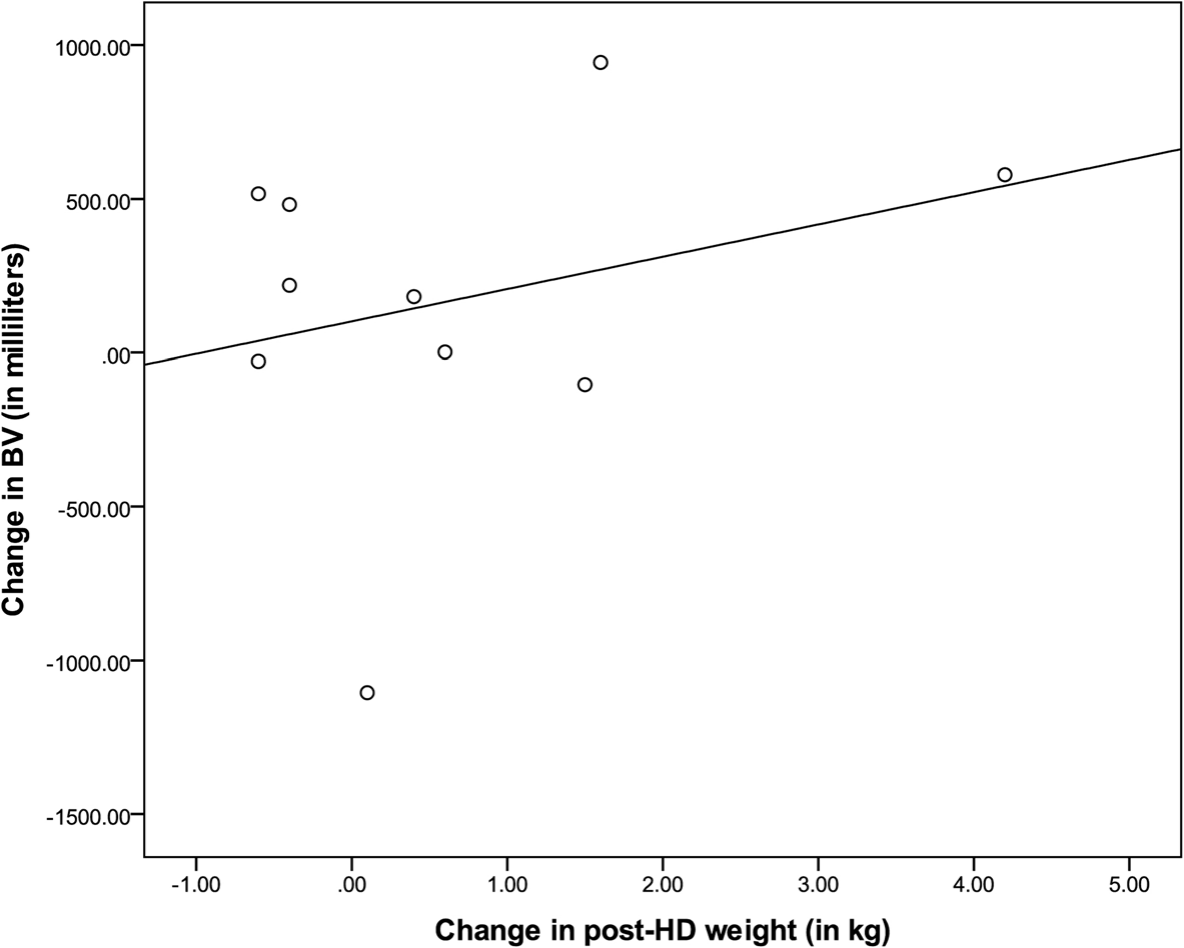
Change in measured BV between 1st and 2nd BVA (in %) according to change in measured post dialysis weight (in kg). Change in measured BV(blood volume) (y-axis) is the difference between measured BV at the second BVA (blood volume analysis measurement) and the BV measured at the first BVA. The change in post-HD (post-hemodialysis) (y-axis) weight equals the post-HD weight on the day of the second BVA minus the post-HD weight on the day of the first BVA. Both the change in measured BV and the change in post-HD weights were calculated for every patient. Linear regression was performed and shows no linear relationship between changes in post-HD weight and changes in measured BV (coefficient of determination, r ^2^ = 0.08)

The difference between post-HD weight and prescribed EDW was similar between patients who had their EDW changed per protocol and those who did not (P value 0.75). There was no linear relationship between the change in absolute post-HD weight and change in BV in our patient group (Fig. 2).

At baseline, the prevalence of intradialytic complications was low in both groups. No changes in the occurrence of intradialytic hypotension, hypertension or cramps occurred in patients regardless of whether a change in EDW was prescribed. Changing the EDW prescription based on BVA did not affect the occurrence of intradialytic complications. As expected from a prior report [32], there was no apparent association between cramping and iatrogenic volume depletion. Two out of the 3 patients with cramps at a particular session were considered euvolemic for that session while the 3rd patient was deemed volume overloaded.

## Discussion

We previously demonstrated that BVA could be performed in stable HD patients and could yield estimates of BV and correlated with CLM-III readings [27]. Interventional trials have also performed BVA to guide fluid management and diuresis in hospitalized patients [22, 25, 26].

This study is a pilot to determine feasibility, potential benefits and reproducibility of volume status estimation for outpatients with ESKD on hemodialysis. Despite its feasibility, BVA did not lead to an improvement in achieving euvolemia in our small patient population of chronic HD patients. On the other side, BVA measurements were stable when comparing the first and second measurement; also, patients who did not have a change in EDW per protocol remained euvolemic according to the repeated measurement.

Flythe et al. recently reported an increased mortality for patients who were more than 2 kg away from prescribed EDW at the end of dialysis sessions [33]; in our study we used a narrower margin and only considered 2 hemodialysis sessions. The difference between EDW and post-HD weight was not affected by changing EDW based on BVA readings and our overall incidence of complication was low as documented in Table 1..

Our protocol was based on the assumption that the observed difference between measured and ideal BV could be extrapolated to a desired change in weight. Based on our data, it is not possible to validate our initial assumption that a change in target weight would affect BV in a linear fashion (Fig. 2). Weight and BV were even found to change in opposite direction in certain cases (Fig. 2). This can be partly explained by the average of 70 days between both measurements, and also by the fact that BVA measures intravascular volume but not total ECV.

Our data are consistent with prior reports showing small changes in BV measurements by BVA in patients with heart failure who were admitted for diuresis despite large changes in body weight upon hospital discharge [26]. Previous BVA data in patients on HD also described a wide variability of the percentage of total weight loss that is in fact from the intravascular component (54 to 99%) [27]. The heterogeneous source of fluid loss can therefore explain the absence of a linear relationship when several patients are considered in this study.

Our current observation is that despite the variability in post-HD weights, BV measurements were reproducible over 1–3 months in patients on chronic hemodialysis. Thus, a cross-sectional BV measurement may provide information about chronic volume status that is an important factor influencing morbidity in the ESKD population [6, 11, 15, 34, 35]. This finding further emphasizes the need to realize that a large proportion of fluid removal occurs from interstitial ECV while intravascular ECV appears to be more stable over time and may require a longer-term strategy to be controlled.

Our study has several limitations related to the small sample size and to the fact that participants were generally not far from what was judged to be a desirable or ideal weight. The technique may be more useful for patients newly initiated on HD. BVA may also provide useful information about intravascular ECV to guide management in patients with intradialytic hemodynamic instability.

The second BVA measurement occurred at variable intervals after the first to suit patient and physician preferences. We viewed this as a pragmatic allowance given that the patients were not unstable. The ideal time interval between the initial reading and the follow up measure is currently indeterminate. BVA requires trained personnel, equipment and availability of a nuclear medicine facility (which are possible to obtain in the outpatient setting). The measurement also requires patient cooperation for a time consuming procedure and consent to be subjected to low doses of radiation. Planning measurements and repeating BVA should balance patient safety, resource allocation and stability of lean body mass (i.e. non-volume-related body weight).

Non-invasive alternatives to BVA have been developed for determination of volume status in hemodialysis patients. While BVA estimates the whole body’s intravascular BV, lung ultrasound evaluates pulmonary congestion and cannot provide information about intravascular fluid or other areas of interstitial fluid accumulation. Inferior vena cava ultrasound measures central vasculature filling and thus only intravascular volume. Bioimpedance spectroscopy (BIS) estimates all extracellular volume and thus cannot appreciate intravascular and interstitial fluid separately. BIS is a promising, non-invasive technique and has been validated as an accurate estimator of extracellular volume against dilution techniques [36, 37]. BIS has been used during HD to with some success to minimize hypotension [38] and improve blood pressure control [8–10].

It is important to keep in mind that BIS and BVA measure different volumes and may be different pieces of the same puzzle to reach “euvolemia”. Many patients with ESRD suffer from chronic edema and probing to normalize their total ECV may lead to depletion of intravascular ECV. BVA may be helpful in quantitatively assess the intravascular fluid compartment in these patients and establish an ideal rather than “dry” weight. Many of our patients had a mild increase in BV from ideal, between 1 to 11% (the normal range is up to 8%) and did not have a high incidence of intradialytic complications.

## Conclusions

Our pilot study demonstrates that in a relatively clinically stable population, BV measurements are stable over 1–3 months. The reproducibility of BV measurements despite changes in post-HD weight suggests that most of the fluid removal in dialysis is ultimately pulled from the interstitium rather than intravascular space. BVA may therefore be used to assess chronic volume status rather than acute changes in the ESKD population. Further studies with a more diverse and larger sample population may still be needed in order to further evaluate the relationship between change in weight and change in BV over time. We would look forward to an adequate randomized controlled trial that would be powered to evaluate changes in morbidity after altering EDW according to BVA results and determine an outcome-based target range for BV. Establishing an outcome-based “normal range” for patients of hemodialysis would make BVA a clinically useful test and shift our goal from probing for “dry weight” to identifying clinically safe BV.

## Abbreviations

BIS: Bioimpedance spectroscopy
BV: Blood volume
BVA: Blood volume analysis
ECV: Extracellular fluid volume
EDW: Clinically prescribed “estimated dry weight”
ESKD: End stage kidney disease
Hct: Peripheral hemactocrit
HD: Hemodialysis
post-HD: Post-dialysis
PV: And plasma volume
VA-NYHHS: New York Harbor Department of Veterans Affairs Healthcare System
